# Antifungal Effects of the Phloroglucinol Derivative DPPG Against Pathogenic *Aspergillus fumigatus*

**DOI:** 10.3390/antibiotics14050499

**Published:** 2025-05-13

**Authors:** Liyang Wang, Junying He, Hanzhong Feng, Qian Li, Meirong Song, Haoran Gou, Yongxing He, Kui Zhu

**Affiliations:** 1 National Key Laboratory of Veterinary Public Health and Safety, College of Veterinary Medicine, China Agricultural University, Beijing 100193, China; sy20193050848@cau.edu.cn (L.W.); sy20233051122@cau.edu.cn (J.H.); liqianjiu@jlu.edu.cn (Q.L.); meirong_song@cau.edu.cn (M.S.); 2 Ministry of Education Key Laboratory of Cell Activities and Stress Adaptations, School of Life Sciences, Lanzhou University, Lanzhou 730000, China; fenghzh19@lzu.edu.cn (H.F.); gouhr2023@lzu.edu.cn (H.G.); heyx@lzu.edu.cn (Y.H.)

**Keywords:** *Aspergillus fumigatus*, phloroglucinol derivative, 2,4-diproylphloroglucinol (DPPG), antifungal activity, membrane homeostasis

## Abstract

**Background:** Fungal infections pose an increasingly predominant threat to human and animal health. Modified compounds derived from chemo-diverse natural products offer enhanced therapeutic efficacies and promising approaches to combat life-threatening fungal pathogens. **Methods:** We performed biosynthetic gene clusters analysis of 2,4-diacetylchloroglucoside (DAPG) in 4292 shotgun metagenomes samples from the healthy and diseased skin. Then, we assessed the antifungal activity of DAPG and the derivative 2,4-diproylphloroglucinol (DPPG) against pathogenic fungi by minimum inhibitory concentrations. The inhibitory effects of DPPG were measured using hyphal growth assay and spore germination assay. Concurrently, the mechanism of DPPG on *Aspergillus fumigatus* was investigated in membrane permeability and fluidity. The therapeutic efficacy was evaluated in a *Galleria mellonella* infection model. **Results:** We observed a significantly higher abundance of bacteria harboring DAPG biosynthetic clusters on healthy skin compared to diseased skin. Further, we designed and synthesized a series of phloroglucinol derivatives based on DAPG and obtained an antifungal candidate DPPG. DPPG not only exhibited robust antifungal activity against *Aspergillus* spp. and *Candida* spp. but also impaired hyphal growth and spore germination of *A. fumigatus* in vitro. A mechanism study showed that DPPG reduced membrane fluidity and increased the leakage of cellular contents, resulting in membrane perturbation and fungal death. Lastly, the therapeutic efficacy of DPPG was confirmed in a *G. mellonella* infection model. **Conclusions:** Our study demonstrates that DPPG is a potent scaffold to combat invasive fungal infections.

## 1. Introduction

Fungal pathogens have emerged as a crucial threat to public health with an annual incidence of 6.5 million infections and 3.8 million deaths [1,2]. Despite the availability of three major classes of antifungal drugs (polyenes, azoles, and echinocandins) to treat systemic fungal infections [3,4,5], drug resistance remains a great challenge, and the course of treatment can be complex. Increasing attention has been focused on novel antifungal chemicals against hazardous fungal pathogens to alleviate the intensifying burden of diseases. *Aspergillus fumigatus*, one of the crucial opportunistic pathogens, is responsible for invasive aspergillosis in humans and animals (e.g., birds) through inhaling spores in the environment [6,7]. *A. fumigatus* can cause various severe diseases (e.g., chronic pulmonary aspergillosis, otitis externa, *Aspergillus* endocarditis, and cutaneous aspergillosis) in both immunocompetent and immunocompromised patients [7]. Demethylation inhibitors, which target fungal cytochrome P450 enzymes, are widely used in both clinical and agricultural settings. However, cross-resistance between them has been demonstrated, leading to resistance to triazole which is the first-line antifungal drugs used to treat aspergillosis [8,9]. Given the high mortality rate and increasing antifungal resistance exhibited by *A. fumigatus*, there is an urgent need to discover novel antifungal agents and optimize clinical therapeutic strategies [10].

The discovery of bioactive molecules derived from natural products has made great contributions to the development of drugs for various purposes [11,12]. For example, amphotericin B, isolated from soil-dwelling *Streptomyces nodosus*, exhibits broad-spectrum efficacy and has been reserved as a last line of defense in clinic [13,14]. Subsequently, based on the active scaffold and antifungal mechanism, the derivatives were carried out with better selectivity and renal-sparing characteristics [15,16]. However, the discovery of natural products with antimicrobial activity is often achieved through microbial interactions. In recent years, fluorescent pseudomonads have received increasing attention as a biocontrol product in agriculture owing to their properties in controlling plant diseases caused by fungal and bacterial pathogens [17,18,19,20,21]. Further studies revealed that 2,4-diacetylchloroglucoside (DAPG) produced by *Pseudomonas fluorescens* has proved to be an important molecule in killing soil-borne plant pathogens including bacteria, fungi, and others [22]. The broad-spectrum activity and biocompatible property made DAPG a promising antimicrobial molecule. Similarly, the skin also provides a home for a variety of commensal microbiota, and interactions between microbiota on the skin are ubiquitous [23]. In a recent study, the commensal *Staphylococcus lugdunensis* on the skin was found to inhibit the growth of pathogenic *Staphylococcus aureus* by producing a novel thiazolidine-containing cyclic peptide [24]. Therefore, the discovery and modification of natural products based on microbiota interactions presents a promising approach for further research.

In this study, we obtained a novel phloroglucinol derivative 2,4-diproylphloroglucinol (DPPG) with modified acyl groups at the positions of C2 and C6, which exhibited broad-spectrum activity against clinically important fungi (e.g., *Candida* spp. and *Aspergillus* spp.). Specifically, DPPG inhibited hyphal growth and spore germination of *A. fumigatus* in a dose-dependent manner, which exerted the antifungal property via membrane perturbation and content leakage. In addition, DPPG also showed therapeutic efficacy against *A. fumigatus* in *Galleria mellonella* model. Our study demonstrates that DPPG is a potent chemical scaffold against fungal pathogens.

## 2. Results

### 2.1. Distribution of DAPG Biosynthetic Clusters in Skin

**Figure 1:**
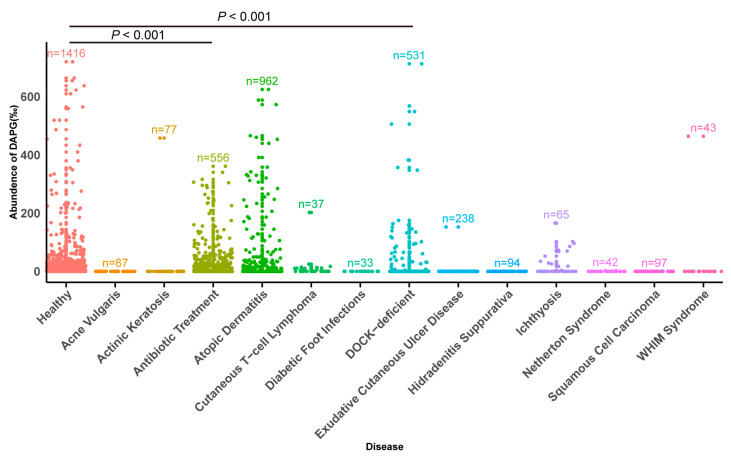
Distribution of the levels of bacteria containing the DAPG biosynthetic clusters across various disease conditions and healthy skin, with the number of samples (*n*) indicated for each condition.

To characterize the colonization dynamics and metabolism changes of microbiota on the skin surface under healthy and diseased states, we analyzed the levels of bacteria containing DAPG biosynthetic clusters and found differences between healthy and diseased skin. Notably, there was a significant reduction in the levels of bacteria containing the DAPG biosynthetic clusters in immunosuppressed skin diseases (e.g., diabetic foot infections and squamous cell carcinoma) (*p* < 0.001). The average percentage reduction was 20.1‰ in both diabetic foot infections squamous cell carcinoma compared with the healthy skin group (Figure 1). The results suggest that DAPG not only plays an important role against plant pathogens but also has the potential to contribute to skin health. Therefore, we hypothesize that DAPG may also possess the ability to antagonize human fungal pathogens.

### 2.2. In Vitro Antifungal Activity of DAPG and DPPG

**Figure 2:**
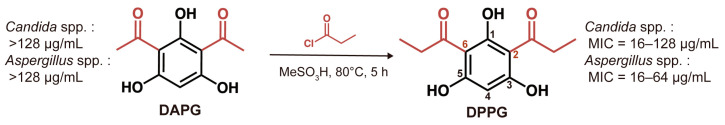
The antifungal activity and detailed reaction equation of DAPG and DPPG.

According to the hypothesis above, we tested the antifungal activity of DAPG against a variety of *Candida* spp. (*C. albicans*, *C. tropicalis*, and *C. krusei*) and *Aspergillus* spp. (*A. fumigatus*, *A. flavus*, *A. niger*, and *A. terrestris*) strains. As shown in Table 1, the activity of DAPG was weak with Minimum Inhibitory Concentrations (MIC) exceeding 128 μg/mL. It has been reported that the length of acyl chain at the positions of C2 and C6 and the position of the phenolic hydroxyl groups could influence the antimicrobial activity [25,26]. Based on the previous study, we synthesized the derivative DPPG and attempted to explore the antifungal activity against pathogenic fungi (e.g., *Candida* spp. and *Aspergillus* spp.) affecting humans to broaden its application. The MICs of DPPG were evaluated and presented in Table 1, respectively. We found that DPPG exhibited notable activity against *Candida* spp. with MIC values of 16–128 μg/mL and against *Aspergillus* spp. with MIC values of 16–64 μg/mL (Supplementary Table S1). Hence, it suggests that the derivative DPPG enhances the antifungal activity and broadens the antifungal spectrum (Figure 2).

### 2.3. Inhibitory Effect on Hyphal Growth of DPPG

**Figure 3:**
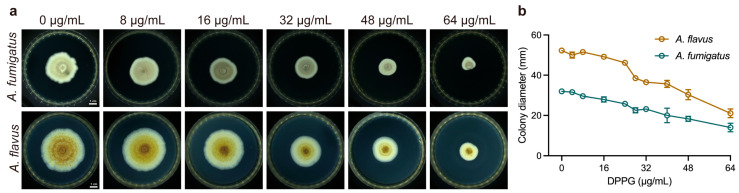
In vitro anti-hyphal effect of DPPG against *A. fumigatus* and *A. flavus*. (**a**) The hyphal growth inhibition of *A. fumigatus* ATCC 96918 and *A. flavus* ATCC 11492 incubated at 30 °C on PDA medium for five days. (**b**) Changes in colony diameters of *A. fumigatus* ATCC 96918 and *A. flavus* ATCC 11492 incubated at 30 °C on PDA medium for five days. The presented data are depicted as means ± deviation (SD). Data represent two biological replicates.

To further characterize the inhibitory effect of DPPG in *Aspergillus* spp. in different physiological states, we measured hyphal growth and spore germination under the treatment of DPPG, respectively. DPPG significantly inhibited the spread of fungal mycelium and densified the mycelium accompanied with pigmentation reduction in a dose-dependent manner (Figure 3a). Meanwhile, DPPG at 32 μg/mL dramatically reduced most of the hyphal growth (Figure 3b), with diameters reduced from 32 cm to 23 cm. These observations indicate that DPPG possesses a potent anti-hyphal activity against *Aspergillus* spp.

### 2.4. Inhibitory Effect on Spore Germination of DPPG

**Figure 4:**
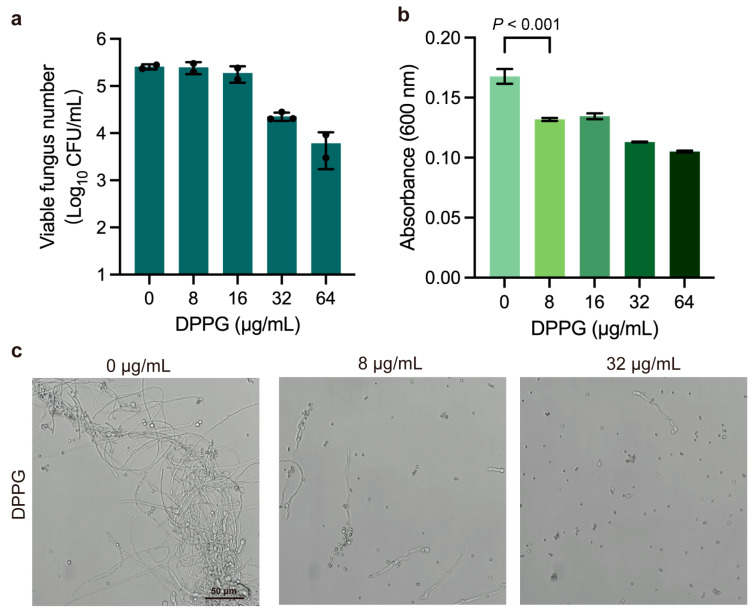
The inhibition of spore germination of DPPG against *A. fumigatus*. (**a**) The viable fungus number in the SDA medium containing DPPG at 37 °C. (**b**) The optical density at 600 nm after DPPG treatment. (**c**) Imaging of *A. fumigatus* spore germination at 37 °C in RPMI1640 culture medium. The presented data are depicted as means ± deviation (SD). Data represent two biological replicates.

Radial growth and germination of aspergilli at 37 °C have been associated with pathogenicity [27]. Next, we also determined spore germination based on conducting imaging and colony-forming unit (CFU) measurement under the treatment with sub-inhibitory concentrations of DPPG. DPPG inhibited fungal spore germination in a dose-dependent manner, leading to a reduction of approximately two orders of magnitude in the number of viable fungi on the plates (Figure 4a). Meanwhile, the decrease in optical density suggested that DPPG inhibited spore germinating to hyphae (Figure 4b). It can also be seen that the growth of branched hyphae decreased with rising DPPG concentration. Almost no hyphae were produced after treatment with DPPG at 32 μg/mL (Figure 4c). These results manifest that DPPG has a good inhibitory effect on spore germination in *A. fumigatus*.

### 2.5. Membrane-Targeting Property of DPPG

**Figure 5:**
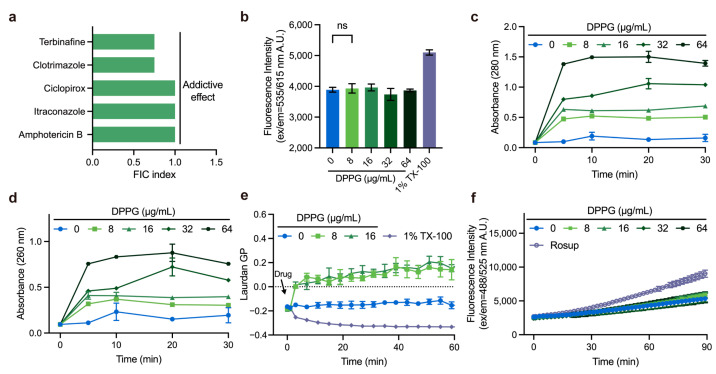
The membrane-targeting property of DPPG. (**a**) Drug interactions of DPPG with a collection of antifungal drugs against *A. fumigatus*. Synergy effect is defined as a FICI ≤ 0.5, addictive effect is defined as a 0.5 < FICI ≤ 1. (**b**) Membrane permeability of *A. fumigatus* after treatment of DPPG in 1 h using propidium iodide. 1% Triton X-100 was used as the positive control. (**c**,**d**) Release of cytoplasmic contents absorbing at 260 nm and 280 nm in *A. fumigatus*. The presented data are depicted as means ± deviation (SD). Data represent two biological replicates. (**e**) Membrane fluidity of *A. fumigatus* after treatment of DPPG. 1% Triton X-100 was used as the positive control. (**f**) ROS accumulation of *A. fumigatus* after DPPG treatment. Rosup at 50 µg/mL was used as the positive control. A *p*-value of “ns” means not significant, suggesting no meaningful difference between groups.

The antifungal properties of DPPG encouraged us to unravel its antifungal mechanism. Synergy study can be suggestive of potential antimicrobial mechanisms. To further assess the potency of DPPG in combination with clinical antifungal agents, we conducted a checkerboard test in combination with a collection of antifungal drugs against *A. fumigatus* ATCC 96918. DPPG manifested the addictive effect with terbinafine, clotrimazole, itraconazole, and ciclopirox with the Fractional Inhibitory Concentration (FIC) index ranging from 0.5 to 1.0 (Figure 5a). Given that addictive effect is found in membrane-targeting agents, we speculated that DPPG had an analogous mechanism with aforesaid antifungal agents. To test this hypothesis, we determined the membrane integrity of *A. fumigatus* under DPPG treatment. The absence of fluorescence enhancement of PI stain suggested that DPPG did not perforate the membrane or disrupt fungal membranes, thereby altering their permeability (Figure 5b). Nevertheless, when treated with DPPG at concentrations ranging from 0 μg/mL to 64 μg/mL, a predominant increase in absorbance at 260 nm and 280 nm was observed within 5 min (Figure 5c,d), denoting the rapid release of intracellular nucleic acids and proteins. These results suggest that there is obvious perturbation on the membrane inflicted by DPPG other than direct pore formation.

To further validate the membrane-targeting mechanism, we determined the physicochemical properties of membrane by measuring the generalized polarization (GP) values of Laurdan-labeled fungi, which were used to evaluate changes in membrane phase properties [28]. Compared to the positive control treated with 1% Triton X-100, the GP value suddenly increased by 0.155 after about 2 min of incubation, suggesting that DPPG possessed a rigidifying effect on fungal membrane (Figure 5e). Finally, we found that DPPG interacted with the fatty acid chains of phosphatidylethanolamine through hydrophobic interaction, providing preliminary evidence that DPPG may cause membrane perturbation by interacting with phospholipids (Supplementary Figure S3).

We also explored the accumulation of ROS during the process of DPPG treatment. To clarify whether inhibitory effect is also directly caused by ROS damage, we determined ROS accumulation using DCFH-DA-labeled fungi (Figure 5f). This demonstrated that DPPG did not accumulate ROS within 1.5 h, unlike conventional antifungal drugs reported in the literature [29].

### 2.6. In Vivo Antifungal Activity of DPPG in G. mellonella Model

**Figure 6:**
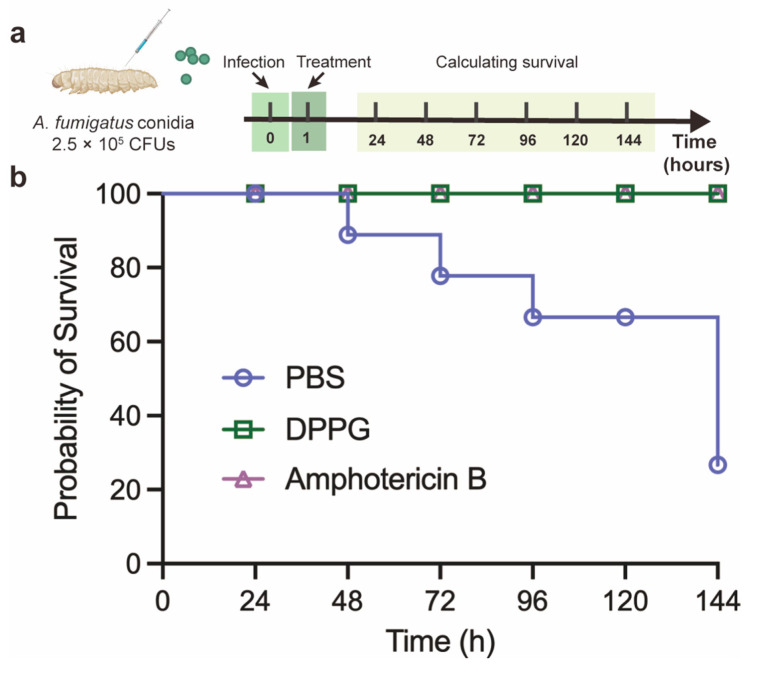
*G. mellonella* infection model. (**a**) Schematic diagram of in vivo antifungal activity using *G. mellonella* model. (**b**) Survival rates of *G. mellonella* larva infected with *A. fumigatus* ATCC 96918 conidia (2.5 × 10^5^ CFUs) after application of PBS, DPPG (10 mg/kg) or amphotericin B (10 mg/kg). *n* = 8 biologically independent animals.

The antifungal performance and relatively low toxicity [25] of DPPG prompted us to figure out the preliminary therapeutic effect in *G. mellonella*. In this model, *G. mellonella* was infected with *A. fumigatus* ATCC 96918 and then treated with PBS, DPPG, and amphotericin B (Figure 6a). As shown in Figure 6b, two out of eight PBS-treated larvae survived; however, the larvae still remained alive after being treated with DPPG and amphotericin B. These results suggest that DPPG (10 mg/kg) has a therapeutic effect comparable to that of amphotericin B (10 mg/kg) in *G. mellonella*.

## 3. Discussion

Secondary metabolites derived from *Pseudomonas fluorescens* strains in the plant rhizosphere, especially the antifungal DAPG, have been well studied [26,30,31,32,33,34,35,36]. Recent studies have also proved its antifungal properties against yeast species [37,38,39]. The structural modification of natural products to obtain novel molecules with higher activity is an important strategy to explore antifungal compounds. Based on the approach of chemical synthesis, we obtained the novel derivative DPPG, which has exhibited robust antibacterial activity against gram-positive bacteria [25]. Therefore, we explored the promising antifungal activity of DPPG to give rise to a broad-spectrum derivative effective against potential pathogens. Our study reveals that DPPG possesses a wide range of activity against *Aspergillus* spp. including multiple clinical isolates. Furthermore, our results provide evidence that DPPG inhibits hyphal growth and spore germination in a dose-dependent manner. Further, antifungal activity of DPPG is exerted through membrane perturbation and cellular content leakage. In addition, DPPG does not induce ROS accumulation to kill the fungi, representing an inhibitory characteristic that is different from that of conventional antifungal drugs. Moreover, the therapeutic efficacy against *A. fumigatus* is also demonstrated in the *G. mellonella* infection model.

The surface of human and animal skin is colonized by a diverse milieu of microorganisms. It is worth noting that the skin is also a reservoir for colonization by *Candida  auris* and *Enterococcus faecium*, *Staphylococcus aureus*, *Klebsiella pneumoniae*, *Acinetobacter baumannii*, *Pseudomonas aeruginosa*, and *Entobacter* species (ESKAPE) pathogens and their associated antimicrobial-resistance genes in a recent study [40]. At the same time, a proportion of cutaneous microbiotas have been observed to produce molecules capable of inhibiting the colonization of other organisms and modulating their behavior [41]. Therefore, it is imperative to understand the interactions between microbial communities, such as the previously mentioned *P. fluorescens* with pathogenic bacteria and fungi in the plant rhizosphere, which may facilitate the exploration of novel antimicrobial compounds. In our research, we found an important natural metabolite DAPG produced by skin bacterial microbiota that decreased significantly in diseased skin, especially immunosuppression related disease. This finding indicated that DAPG may play a pivotal role in skin microbiota. Notwithstanding the fact that the activity of DAPG against human pathogenic fungi was found to be minimal, the structural modification of DAPG, as previously demonstrated in our study, exhibited noteworthy antifungal properties against *Aspergillus* app. This finding provides a solid foundation for the subsequent development of antimicrobial compounds.

As an airborne opportunistic fungal pathogen, *A. fumigatus* may lead to invasive aspergillosis by inhaling spores into the lungs [42]. The physical characteristics (e.g., small size, melanin, and negatively charged cell wall) allow conidia to attach to distal respiratory tract. Also, the thermotolerance property facilitates the growth of conidia in the respiratory tracts of mammals or birds, leading to germinate for invasion [43]. Meanwhile, radial growth and germination rates of aspergilli at 37 °C are associated with pathogenicity [27]. Consequently, antifungal molecules that can inhibit both spore germination and hyphal growth held considerable promise for clinical applications. In our study, the phloroglucinol derivative DPPG exerted an inhibitory effect on both processes against *A. fumigatus* in vitro. DPPG at 32 μg/mL exhibited nearly complete inhibition of spore germination in addition to substantial inhibition of hyphal growth. This observation signifies the potential for further investigation of antifungal mechanism.

Recent studies have illuminated the antifungal activity of DPPG against plant fungal pathogens (e.g., *Botrytis cinerea* and *Monilinia fructicola*) [44], whereas the activity and mechanism against human pathogenic fungi remain unexplored. Consequently, we conducted a tentative study on the inhibitory effect and preliminary mechanisms to enrich antifungal chemical skeleton library. DPPG has been reported to block electron transfer in *S. aureus* by competing with menaquinone (MK) and thus binding to NDH-2 of electron transport chain located in the membrane [25]. In humans and fungi, coenzyme Q (ubiquinone or CoQ) acts as a quinone in the electron transport chain [45]. The negative result (Figure S2) indicated the different targets of DPPG in *A. fumigatus* and inspired us to investigate other alternative mechanisms. In our study, we also found that DPPG does not induce the ROS accumulation in *A. fumigatus*, which is consistent with a previous study in *S. aureus* [25]. Recent studies have proved that reactive oxidant species (ROS) in fungal cells will form in response to the trigger of various types of environmental stress as well as antifungal agents [29]. It is generally accepted that the increment of ROS accumulation arising from antifungal drugs (e.g., polyenes, azoles, and echinocandins) is correlated with fungicidal effect leading to cellular macromolecule damage [29] and it might explain the low rate of resistance to antifungal drugs [46]. Our results reveals a different phenotype compared to conventional antifungal drugs encouraging further exploration of possible mechanisms of this type of molecule without ROS accumulation in the future. However, an important direction for future research is to determine the physiological changes and mode of action within the fungal cell. The mechanism after which the compounds penetrate the fungal membrane also needs to be explored in depth. In addition, modified compounds with enhanced antifungal activity should be designed based on the promising inhibitory effect.

In conclusion, we find that DPPG exhibits a promising antifungal effect against fungal pathogens and preliminarily investigates its mechanism of disrupting membrane homeostasis and content leakage. Our study presents that DPPG is a potent chemical scaffold against torturous pathogens.

## 4. Materials and Methods

### 4.1. Materials and Chemicals

Roswell Park Memorial Institute (RPMI) 1640 culture medium and Tween 20 were purchased from Thermo Fisher Scientific Technology Co., Ltd. (Beijing, China). Phosphate buffered saline (PBS), 4-Morpholinepropanesulfonic acid (MOPS), amphotericin B, terbinafine, clotrimazole, ciclopirox, itraconazole, and nystatin were purchased from Aladdin Biochemical Technology Co., Ltd. (Shanghai, China). Potato dextrose agar (PDA) and Sabouraud agar (SDA) were purchased from Solarbio Science & Technology Co., Ltd. (Beijing, China). Laurdan and propidium iodide were purchased from MedChemExpress Co., Ltd. (Shanghai, China). Triton X-100 and 2′,7′-dichlorodihydrofluorescein diacetate (DCFH-DA) were purchased from Beyotime Biotechnology Co., Ltd. (Shanghai, China).

### 4.2. Biosynthetic Gene Clusters Analysis

We analyzed 4292 shotgun metagenomes samples including healthy skin group and 13 pathological cohorts, using an adapted Daniel Dar et al. pipeline [47]. Quality-filtered reads were processed through DIAMOND (v0.9.14) (-k 1 mode) against a BGC reference database, discarding matches with <80% amino acid identity. Gene abundances were normalized by length (reads/kb), with BGC scores derived from median gene values per cluster. DAPG-specific scores were aggregated across homologous BGCs, then normalized against a bacterial biomass index calculated from 16 conserved marker genes. Final relative abundance represented the ratio of aggregated DAPG-BGC scores to total bacterial biomass. Statistical analysis was performed by Wilcoxon rank-sum test.

### 4.3. Fungal Strains and Culture Conditions

*A. fumigatus* ATCC 96918, *A. flavus* ATCC 11492, *C. albicans* ATCC 10231, and *C. krusei* ATCC 6258 were obtained from Guangdong Microbial Culture Collection Center (GDMCC) in China. *C. krusei* CGMCC 2.3984 was obtained from China General Microbiological Culture Collection Center (CGMCC). Other fungal strains were isolated from patients in hospital. All strains were cultured in SDA medium at 30 °C under standard conditions.

### 4.4. Synthesis of the Derivative DPPG

The synthesis procedure was referred to the previous study [25] of our lab. In short, we added phloroglucinol to a solution of alkyl chloride and mesylate acid at 0 °C and heated the reaction to 80 °C with stirring for 5 h. After that, the solution was poured into ice water and simultaneously extracted with ethyl acetate. The extraction was then rinsed with NaHCO_3_ and vacuum-dried with anhydrous Na_2_SO_4_. With assistance of column chromatography, we purified the crude product with dichloromethane/MeOH (100:1, *v*/*v*). Finally, the phloroglucinol analogs DPPG was obtained. The NMR spectrum of DPPG is provided in Figure S1.

### 4.5. Preparation of Spore Suspension

*Aspergillus* spp. was incubated on SDA medium at 30 °C for three days until good sporulation was obtained. Then, suspension was collected by covering colonies with 0.85% saline containing 0.1% Tween 20 and gently scraping the colonies with a pipette. The mixture of hyphal fragments and conidia was allowed to settle for 15 min at room temperature and then the suspensions were transferred to a sterile tube.

### 4.6. Antimicrobial-Susceptibility Test

The broth microdilution assay was referred to the Clinical and Laboratory Standards Institute (CLSI) document M38-A2, Reference Method for Broth Dilution Antifungal Susceptibility Testing of Filamentous Fungi [48]. In brief, we first prepared dilutions of DPPG stock solution with cultural medium (RPMI 1640 buffered with 0.165 mol/L MOPS) and inoculated 100 μL solutions. Good sporulation was induced on PDA medium at 35 °C for 72 h. Conidia were then collected with approximately 3 mL of 0.85% saline containing 0.1% Tween 20. The mixture was left to settle for 20 min, after which the suspensions were transferred to tubes. The density was adjusted to an absorbance of 0.09–0.13 at 530 nm. We then diluted the suspension in the RPMI 1640 culture medium at a ratio of 1:50 (0.4 × 10^4^ to 5 × 10^4^ CFU/mL) and inoculated each well with 100 μL of the solutions. Also, *C. krusei* ATCC 6258 was utilized as quality control in this experiment. Experiments were performed in triplicate. The broth microdilution tray was incubated at 35 °C for 46–50 h. The MIC was interpreted as the lowest concentration of compounds that prevented a reduction in growth of approximately 90% or more compared to the drug-free wells.

### 4.7. Checkerboard Assay

Firstly, 100 μL of RPMI 1640 culture medium was added to a 96-well plate. To prepare the diluted compounds, antifungal drugs (amphotericin B, terbinafine, clotrimazole, ciclopirox, itraconazole, and nystatin) were added in a row and subsequently diluted along the vertical axis. Then, DPPG was added in the first column and then diluted. To prepare inoculum, *A. fumigatus* was incubated in the PDA medium at 35 °C for 3 d and spores were collected with 0.85% saline containing 0.1% Tween 20. After adjusting the absorbance, the 1:50 inoculum dilutions were prepared in RPMI 1640 culture medium and were twofold at approximately 0.4 × 10^4^ to 5 × 10^4^ CFU/mL. Finally, 100 μL of spore suspension was added except negative control. After incubation for 46–50 h at 35 °C, the MICs were interpreted.

The fractional inhibitory concentration (FIC) index was calculated in accordance with the following formula:

FIC index (FICI) = MIC_ab_/MIC_a_ + MIC_ba_/MIC_b_ = FIC_a_ + FIC_b_ (1)

MIC_a_ and MIC_b_ are the MICs of compound a and b, respectively; MIC_ab_ is the MIC of compound a in combination with compound b; MIC_ba_ is the MIC of compound b in combination with compound a. FICI ≤ 0.5: synergistic effect; 0.5 < FICI ≤ 1: additive effect; 1 < FICI ≤ 4: indifference effect; FICI > 4: antagonism effect.

### 4.8. Hyphal Growth Assay

Hyphal growth of *A. fumigatus* and *A. flavus* was determined under a variety of concentrations of DPPG treatments. Briefly, mycelial blocks of 6 mm diameter were collected, and spores were removed as far as possible. The blocks were clung closely on the center of PDA medium containing DPPG (final concentrations of 0 μg/mL, 4 μg/mL, 8 μg/mL, 16 μg/mL, 24 μg/mL, 28 μg/mL, 32 μg/mL, 40 μg/mL, 48 μg/mL, and 64 μg/mL) and incubated at 30 °C for five days. Experiments were performed in triplicate. Colony diameters were assessed using the criss-cross method [49].

### 4.9. Spore Germination Assay

Spore germination of *A. fumigatus* was measured according to a previous study [50] with minor modifications. Succinctly, we resuspended the spore solutions in RPMI 1640 culture medium containing DPPG (final concentrations of 0 μg/mL, 8 μg/mL, 16 μg/mL, 32 μg/mL, and 64 μg/mL) and incubated them at 35 °C for 24 h. Spore germination was observed using a confocal laser scanning microscope (400 × magnification, Leica TCs SP8 Multiphoton Microscope, Mannheim, Germany). Meanwhile, 100 μL of resuspended spores was also incubated in SDA medium containing DPPG (final concentrations of 0 μg/mL, 8 μg/mL, 16 μg/mL, 32 μg/mL, and 64 μg/mL) for mycelial counting.

### 4.10. Membrane Integrity Assays

The effect of fungal membrane integrity was detected using PI staining referring to a previous study [51]. In a few words, spores of *A. fumigatus* were collected and diluted to A530 = 0.13. Then, spores were washed with PBS buffer after incubation at 37 °C for 24 h. A PI probe (final concentration of 30 µM) was added and stained for 30 min. DPPG solutions (final concentrations of 0 μg/mL, 8 μg/mL, 16 μg/mL, 32 μg/mL, and 64 μg/mL) were added, and fluorescence (Ex/Em = 535 nm/615 nm) was measured using a microplate reader (TECAN Infinite M Plex, Mennedorf, Switzerland) within 1.5 h at 37 °C. A total of 0.1% Triton X-100 was used as a positive control.

### 4.11. Cytoplasmic Content Leakage Test

According to the described method [52] with partial modification, we incubated *A. fumigatus* on SDA medium for good germination and collected spores for 24 h at 37 °C. DPPG solutions (final concentrations of 0 μg/mL, 8 μg/mL, 16 μg/mL, 32 μg/mL, and 64 μg/mL) were added into the medium, and 100 μL of the suspensions was collected for measurement of A260 and A280, respectively. Experiments were performed in triplicate.

### 4.12. ROS Measurement

ROS accumulation was detected using DCFH-DA with a slight modification of the reference method [52]. Spores of *A. fumigatus* were collected as described above and incubated at 37 °C for 24 h. Then, spores were washed with PBS buffer. DCFH-DA (final concentration of 10 µM) was added and stained for 30 min. A total of 50 µg/mL of Rosup was used as a positive control. DPPG (final concentrations of 0 μg/mL, 8 μg/mL, 16 μg/mL, 32 μg/mL, and 64 μg/mL) were added, fluorescence values (Ex/Em = 488 nm/525 nm) were measured with a microplate reader (TECAN Infinite M Plex, Mennedorf, Switzerland) within 1.5 h at 37 °C.

### 4.13. G. Mellonella Infection Model

The therapeutic efficacy of DPPG in *G. mellonella* was evaluated with reference to a previous study [25,53]. The larvae of *G. mellonella* (280 mg per larvae) were pre-incubated at room temperature in advance, after which they were divided into three groups (eight larvae per group). A total of 10 μL of *A. fumigatus* ATCC 96918 (2.5 × 10^5^ CFUs) spores were injected into the right posterior prolegs. After 1 h post-infection, the larvae were injected with amphotericin B (10 mg/kg), DPPG (10 mg/kg) and PBS solution into the left posterior prolegs. The number of alive larvae was recorded and assessed as the survival rate of *G. mellonella* every 24 h at 37 °C.

### 4.14. Molecular Docking

Molecular docking study was performed to predict the binding of membrane lipids (ergosterol and phosphatidylethanolamine) with DPPG by the CDDOCKER module using Discovery Studio 2019 Client (v19.1.0.18287). The structures of fungal membrane lipids referred to a previous study [54].

### 4.15. Statistical Analysis

Statistical analyses were performed using Prism 10 (GraphPad Software, San Diego, CA, USA) and one-way ANOVA was used to calculate significant differences. Data were calculated as mean ± standard deviation of each response variable. *p* < 0.05 indicated a statistically significant difference. Biosynthetic clusters of bacteria were analyzed using the MIBiG database.

## Figures and Tables

**Table 1:** Antifungal activity of DAPG and DPPG against *Candida* spp. and *Aspergillus* spp.

Strains	Origin	MIC ^1^ (μg/mL)	
		DAPG	DPPG
*A. fumigatus* ATCC 96918	Standard strain	>128	32
*A. fumigatus* CAUF136	Human, respiratory infection	>128	32
*A. flavus* ATCC 11492	Standard strain	>128	64
*A. flavus* CAUF135	Human, respiratory infection	>128	32
*A. niger* CAUF137	Human, respiratory infection	>128	16
*A. niger* CAUF140	Human, respiratory infection	>128	32
*A. terrestris* CAUF138	Human, respiratory infection	>128	16
*A. terrestris* CAUF139	Human, respiratory infection	>128	16
*C. albicans* ATCC 10231	Standard strain	>128	64
*C. albicans* CAUF1	Human, urinary tract infection	>128	128
*C. albicans* CAUF3	Human, intestinal infection	>128	128
*C. tropicalis* CAUF2	Human, urinary tract infection	>128	64
*C. tropicalis* CAUF5	Human, intestinal infection	>128	64
*C. krusei* ATCC 6258	Standard strain	>128	16
*C. krusei* CGMCC 2.3984	Standard strain	>128	32

^1^ MICs are interpreted as the lowest concentration of compounds that prevents approximately 90% or more reduction in growth compared to the drug-free wells.

## Data Availability

All data are contained within this article.

## Supplementary Materials

The following supporting information can be downloaded at: https://www.mdpi.com/article/10.3390/antibiotics14050499/s1, Table S1: Antifungal activity of DPPG and amphotericin B. Figure S1: The NMR spectra of DPPG. Figure S2: Exogenous addition of CoQ_10_. Figure S3: Molecular docking of DPPG with fungal membrane phosphatidylethanolamine (PE).

## Abbreviations

DPPG	2,4-diproylphloroglucinol
DAPG	2,4-diacetylchloroglucoside
RPMI	Roswell Park Memorial Institute
MOPS	4-Morpholinepropanesulfonic acid
PBS	phosphate buffered saline
PDA	potato dextrose agar
SDA	sabouraud agar
DCFH-DA	2′,7′-dichlorodihydrofluorescein diacetate
PI	propidium iodide
ROS	reactive oxidant species
CLSI	Clinical and Laboratory Standards Institute
FIC	fractional inhibitory concentration
MIC	minimum inhibitory concentration
CFU	colony-forming unit
GP	generalized polarization
BGC	biosynthetic gene cluster
