# Supplementary material for: Antifungal Effects of the Phloroglucinol Derivative DPPG Against Pathogenic Aspergillus fumigatus
Source: Antibiotics (Basel). 2025 May 13;14(5):499. doi: 10.3390/antibiotics14050499 (PMC12108449; doi:10.3390/antibiotics14050499)
Supplement: Supplementary file 1 [file antibiotics-14-00499-s001.zip › antibiotics-3574587-supplementary.pdf]

# Supporting Information

# Antifungal effects of the phloroglucinol derivative DPPG against pathogenic *A. fumigatus*

**Liyang Wang [1], Junying He [1], Hanzhong Feng [2], Qian Li [1], Meirong Song [1], Haoran Gou [2], Yongxing He [2] and Kui Zhu [1, *]**

1 National Key Laboratory of Veterinary Public Health and Safety, College of Veterinary Medicine, China Agricultural University, Beijing 100193, China; sy20193050848@cau.edu.cn (L.W.); sy20233051122@cau.edu.cn (J.H.); liqiancau@163.com (Q.L.); meirong_song@cau.edu.cn (M.S.).

2 Ministry of Education Key Laboratory of Cell Activities and Stress Adaptations, School of Life Sciences, Lanzhou University, Lanzhou 730000, China; fenghzh19@lzu.edu.cn (H.F.); gouhr2023@lzu.edu.cn (H.G.); heyx@lzu.edu.cn (Y.H.)

* Correspondence: zhuk@cau.edu.cn (K.Z.)

Table S1 Antifungal activity of DPPG and amphotericin B

| Strains | MIC [1] (μg/mL) | |
|---|---|---|
| | DPPG | Amphotericin B |
| *Aspergillus fumigatus* ATCC 96918 | 32 | 1 |
| *Aspergillus flavus* ATCC 11492 | 64 | 2 |
| *Candida albicans* ATCC 10231 | 64 | 1 |
| *Candida krusei* ATCC 6258 | 16 | 2 |

[1] Minimal inhibitory concentration

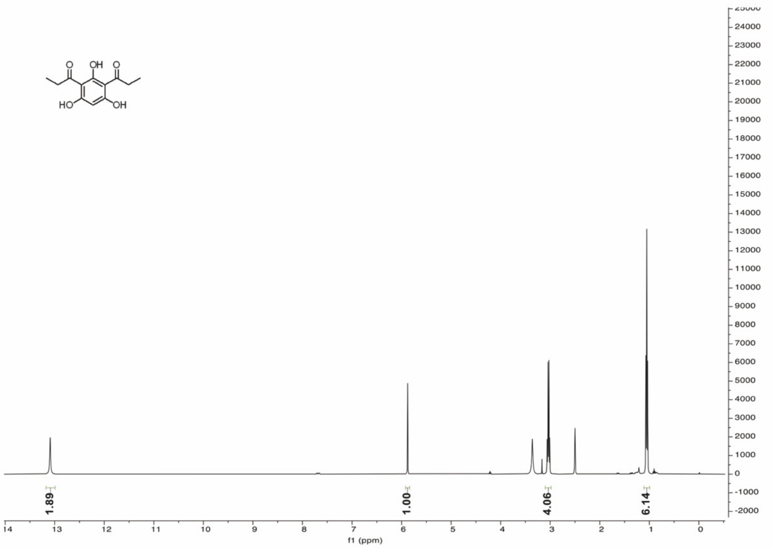


**¹H NMR (400 MHz, DMSO-*d*$_6$)** δ 13.09 (s, 2H), 5.88 (s, 1H), 3.04 (d, *J* = 7.2 Hz, 4H), 1.06 (d, *J* = 7.2 Hz, 6H).

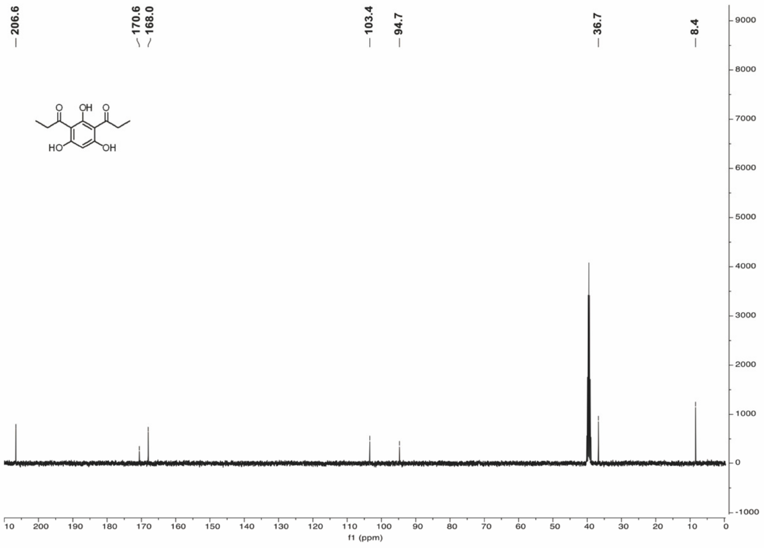


**$^{13}$C NMR (101 MHz, DMSO-d$_6$)** δ 206.6, 170.6, 168.0, 103.4, 94.7, 36.7, 8.4.

**Figure S1.** The NMR spectra of DPPG.

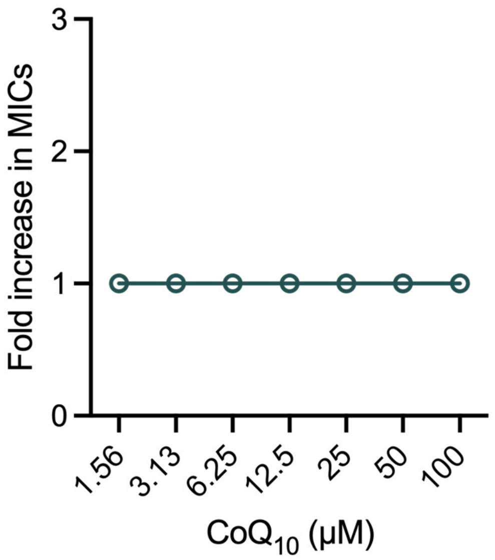


**Figure S2.** Exogenous addition of $CoQ_{10}$. Coenzyme $Q_{10}$ was added to the broth and the antifungal activity of DPPG against *A. fumigatus* ATCC 96918 was performed.

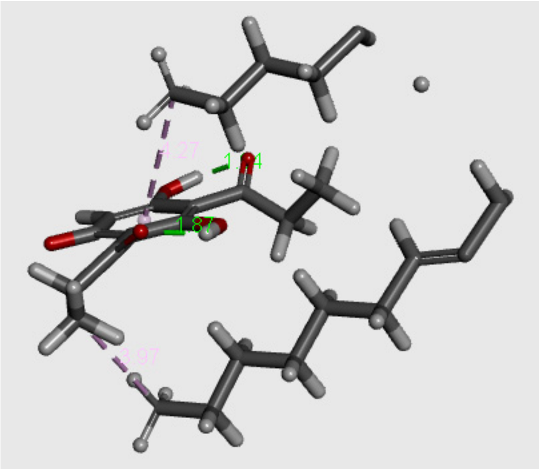


**Figure S3.** Molecular docking of DPPG with fungal membrane phosphatidylethanolamine (PE). The CDOCKER interaction energy of DPPG and PE was –9.21 kcal/mol. The main type of interaction was hydrophobic interaction.
